# Phase 1b trial of anti‐HER2 antibody inetetamab and pan‐HER inhibitor pyrotinib in HER2‐positive advanced lung cancer

**DOI:** 10.1002/mco2.536

**Published:** 2024-04-29

**Authors:** Yihua Huang, Yuanyuan Zhao, Yan Huang, Yunpeng Yang, Yaxiong Zhang, Shaodong Hong, Hongyun Zhao, Shen Zhao, Ting Zhou, Gang Chen, Huaqiang Zhou, Yuxiang Ma, Ningning Zhou, Li Zhang, Wenfeng Fang

**Affiliations:** ^1^ Department of Medical Oncology State Key Laboratory of Oncology in South China Guangdong Provincial Clinical Research Center for Cancer Collaborative Innovation Center for Cancer Medicine Sun Yat‐Sen University Cancer Center Guangzhou PR China; ^2^ Department of Clinical Research State Key Laboratory of Oncology in South China Guangdong Provincial Clinical Research Center for Cancer Collaborative Innovation Center for Cancer Medicine Sun Yat‐sen University Cancer Center Guangzhou PR China

**Keywords:** HER2 mutations, inetetamab, non‐small cell lung cancer, pyrotinib

## Abstract

There remains an unmet need for targeted therapies against advanced non‐small‐cell lung cancer (NSCLC) with HER2 mutations. To improve the antitumor activity of single anti‐HER2 agent, this prospective, single‐arm clinical trial (NCT05016544) examined the safety profile and efficacy of anti‐HER2 antibody inetetamab and pan‐HER TKI pyrotinib in HER2‐posivite advanced NSCLC patients. Enrolled patients received inetetamab every 3 weeks and pyrotinib once per day (pyrotinib, dose‐escalation part, 240 mg, 320 mg; dose‐expansion part, 320 mg). Primary endpoints were dose‐limiting toxicity (DLT) dosage and safety. Secondary endpoints included progression‐free survival (PFS), objective response rate (ORR), and disease control rate (DCR). A total of 48 patients were enrolled. During the dose‐escalation period, no DLT occurred. Diarrhea was the most commonly reported treatment‐related adverse event (TRAE). Grade 3 TRAEs occurred in seven patients. The median PFS (mPFS) was 5.5 months [95% confidence interval (CI): 4.4–8.6 months]. The confirmed ORR and DCR reached 25% (11/44) and 84.1% (37/44), respectively. Responses were shown in patients with distinct HER2 aberrations. In summary, inetetamab in combination with pyrotinib demonstrated acceptable safety and antitumor activity among patients with advanced HER2‐mutant NSCLC.

## Graphical abstract

Manageable safety and clinical benefit of inetetamab plus pyrotinib were shown in advanced HER2‐mutant NSCLC. Responses were observed in patients with various HER2 aberrations including HER2 exon 20 insertion mutations, missense mutations, and HER2 amplification.

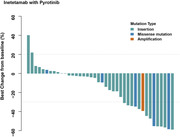

## 1 INTRODUCTION

Lung cancer remains the leading cause of global cancer mortality.^1^ Non‐small‐cell lung cancers (NSCLCs) account for approximately 85% of malignant lung cancer, and the majority of NSCLCs are driven by oncogenic alterations. The discovery of oncogenic alterations and the development of personalized therapies have revolutionized the routine therapies of lung cancer, contributing to improved patient outcomes.^2^, ^3^ Human epidermal growth factor receptor 2 (ERBB2, HER2) mutations comprise 3% of lung cancers and are associated with never‐smoker status, female sex, and adenocarcinoma histology.^4^, ^5^, ^6^ To date, traditional chemotherapy remains the standard‐of‐care for advanced NSCLC with HER2 mutations, with a modest median progression‐free survival (mPFS) of 4.3–6 months.^7^ Despite advances in personalized medicine over the past decades, limited anti‐HER2 agents are available for NSCLC patients harboring HER2 mutations. Effective targeted therapies for these patients are urgently needed.

Clinical efficacies of different HER2‐targeted therapies in advanced NSCLC patients harboring HER2 aberrations have been investigated. Limited clinical activity of currently approved pan‐HER tyrosine kinase inhibitors (TKIs), such as afatinib, neratinib, and dacomitinib has been observed, resulting in an objective response rate (ORR) of 0%–19%.^8^, ^9^, ^10^ Much effort has been devoted to developing anti‐HER2 agents, and improved antitumor effects have been reported. Poziotinib, an irreversible EGFR/HER2 inhibitor, produced an ORR of 27% [95% confidence interval (CI): 12%–46%], with an mPFS of 5.5 months [95% CI: 4.0–7.0 months] in NSCLC patients with HER2 exon 20 mutations.^11^ In the ZENITH20‐2 trial, the ORR of poziotinib reached 27.8% [95% CI: 18.9%–38.2%], and the mPFS was 5.5 months [95% CI: 3.9–5.8 months] in previously treated patients with HER2 exon 20 insertion NSCLC.^12^ However, the high risk of treatment‐related adverse events (TRAEs), mainly grade ≥3, can be a challenge. Pyrotinib is an irreversible inhibitor targeting HER1, HER2, and HER4. Recent studies have demonstrated that pyrotinib could produce manageable toxicity profile and certain clinical benefits, with objective responses occurring in 19.2%–30% of HER2‐mutant lung cancer patients.^13^, ^14^ In a phase II basket trial, ado‐trastuzumab emtansine (TDM1), a HER2‐targeted antibody–drug conjugate (ADC) has shown an ORR of 44% [95% CI: 22%–69%] in HER2‐positive NSCLC patients.^15^ Trastuzumab deruxtecan (T‐DXd, formerly DS‐8201), an anti‐HER2 ADC, showed a 55% ORR [95% CI: 44%–65%] and an mPFS of 8.2 months [95% CI: 6.0–11.9 months].^16^ Based on its superior antitumor activity, T‐DXd was approved for previously treated NSCLC harboring HER2 mutations. Although T‐DXd has manifested the best clinical efficacies in previously treated HER2‐positive lung cancer, it is the only approved HER2‐targeted drug and the TRAEs driven by T‐DXd have a high incidence of grade ≥3 TRAEs (46%) and interstitial lung disease (26%).^16^ Therefore, effective drugs with minor toxicity are still urgently needed to optimize the clinical management of HER2‐mutated NSCLC patients.

Promising efficacies of dual HER2‐targeted regimen have been demonstrated in HER2‐positive breast cancers. Pertuzumab in combination with trastuzumab and chemotherapy has been approved in the neoadjuvant setting for patients with early HER2‐positive breast cancer.^17^ Recent studies also showed the clinical efficacy of neoadjuvant pyrotinib, trastuzumab, and chemotherapy in HER2‐positive breast cancer, further supporting the feasibility of dual HER2‐targeted regimen.^18^ Moderate clinical benefit and manageable toxicity profile of pyrotinib have been demonstrated in advanced NSCLC with HER2 mutations. Combined with positive findings of studies in breast cancer, dual HER2‐targeted therapy might be a promising strategy to further improve the effect of pyrotinib on lung cancer. Inetetamab, a humanized, recombinant anti‐HER2 monoclonal antibody, binds domain IV of HER2 receptor.^19^ Based on the manageable toxicity and antitumor effects shown in clinical trials, inetetamab in combination with vinorelbine has been approved for HER2‐positive metastatic breast cancer by National Medical Products Administration (NMPA) in China.^20^, ^21^ Accumulating studies have investigated the effect of inetetamab combined with pan‐HER TKIs. Preclinical models have demonstrated the synergistic antitumor effects induced by pan‐TKIs in combination with inetetamab.^22^ A phase II clinical trial has shown the encouraging clinical activity and favorable safety of inetetamab in combination with pyrotinib and vinorelbine in HER2‐positive patients with advanced breast cancer.^23^ The preliminary data of a phase II clinical study also demonstrated the clinical antitumor activity and tolerable toxicity of inetetamab, pyrotinib, and chemotherapy in pretreated patients with HER2‐positive metastatic breast cancer.^24^ Although the efficacy of inetetamab was explored in breast cancer, studies on lung cancer are scarce. In addition to the efficacy seen in breast cancer, the higher accessibility of inetetamab in China also facilitates the development of relevant clinical trials. Currently, targeted therapies for patients with HER2‐positive NSCLC remain unmet clinically. Whether dual HER2 blockade consisting of inetetamab and pyrotinib could exhibit acceptable toxicity and more potent antitumor effects in lung cancer remains unclear. This clinical trial was conducted to investigate the safety profile and efficacy of this novel combination therapy in advanced lung cancer patients with HER2 aberrations.

## 2 RESULTS

### 2.1 Patient characteristics

**FIGURE 1:**
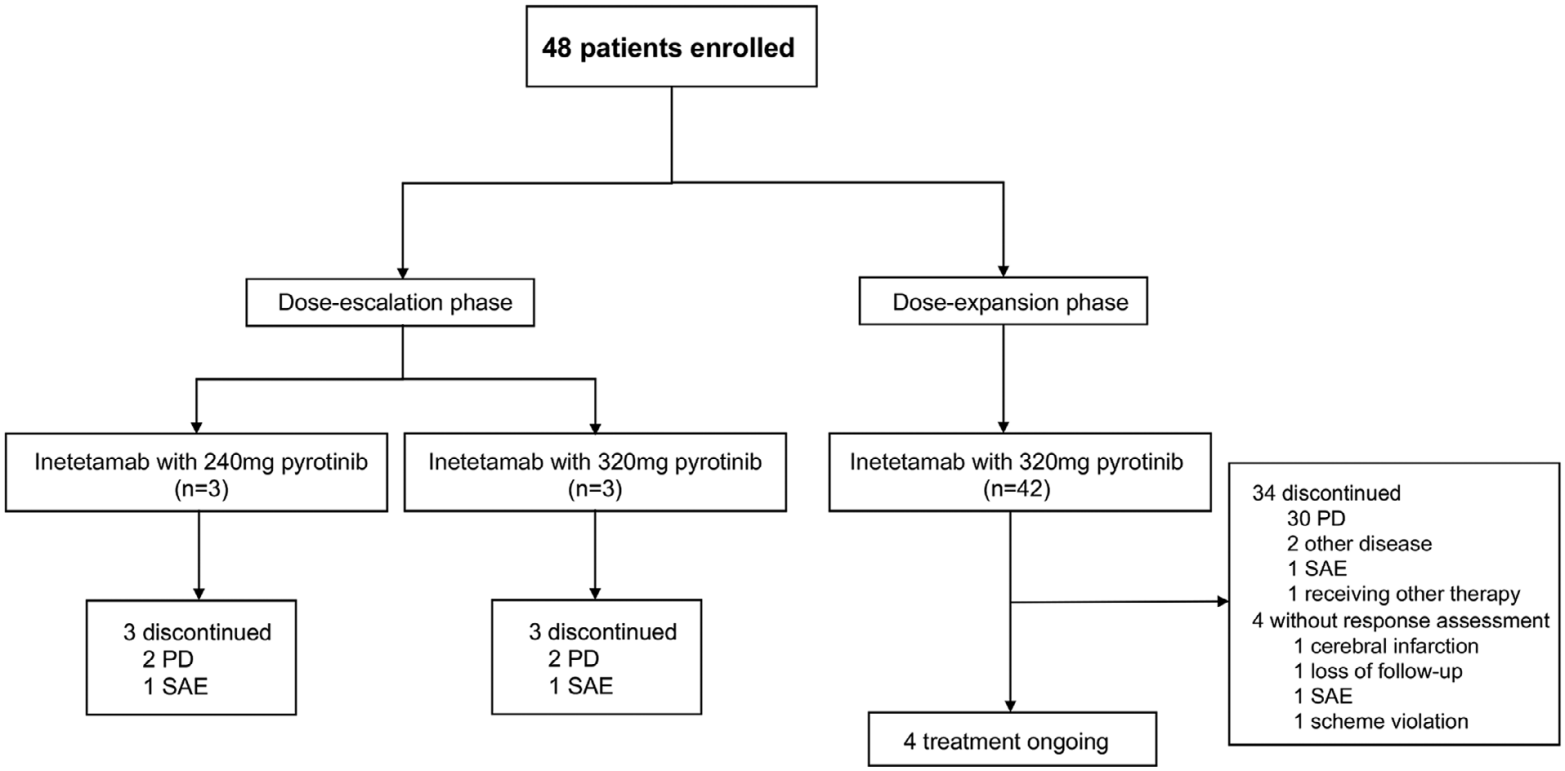
Trial profile. PD, progressive disease; SAE, serious adverse events.

From August 6, 2021 to August 25, 2022, 48 NSCLC patients were enrolled to receive the dual HER2‐targeted combination therapy. During the dose‐escalation period, both inetetamab with 240 mg pyrotinib group and 320 mg pyrotinib group enrolled three patients. During the dose‐expansion phase, 42 patients entered inetetamab with 320 mg pyrotinib group (Figure 1). Safety assessments were performed in all 48 patients receiving at least one cycle of the combination treatment. Forty‐four patients with at least one efficacy assessment were included for efficacy analyses (four patients were excluded for no response assessment). For the efficacy cohort, as of March 20, 2023, four patients remained on study treatment, and 40 discontinued combination therapy (34 due to disease progression, two due to other diseases, three due to grade 3 serious adverse events [SAE], and one due to receiving other antitumor therapies) (Figure 1). Baseline clinical features are summarized in Table 1. The median follow‐up duration was 10 months. The median age of the patients was 55 years (range: 32–74). The majority of them were non‐smokers (41/48, 85.4%) and had stage IV disease (46/48, 95.8%). All patients had adenocarcinoma. Approximately 68.8% (33/48) of the patients had Eastern Cooperative Oncology Group (ECOG) scores of 1. Half of the patients (24/48, 50%) had baseline brain metastases. Twenty‐three patients (23/48, 47.9%) received at least one prior antitumor treatment. Forty‐one patients carried HER2 exon 20 insertion mutations (25 Y772_A775dup, 7 G776delinsVC, 6 G778_P780dup, and three other types), six carried missense mutation (S310F, S310Y/D769H, V659D, V659E, I767M, V777L), and one carried HER2 amplification (copy number 48.8). TP53 status was detected in 45 patients, among whom 27 (27/45, 60%) had positive TP53 aberrations.

**TABLE 1:** Baseline characteristics.

Characteristic	Patients (*N* = 48)
Age, years	
Median (range)	55 (32–74)
Sex, *n* (%)	
Male	23 (47.9)
Female	25 (52.1)
Histology, *n* (%)	
Adenocarcinoma	48 (100)
Stage, *n* (%)	
IIIB	2 (4.2)
IV	46 (95.8)
Brain metastases, *n* (%)	
No	24 (50.0)
Yes	24 (50.0)
Smoking status, *n* (%)	
Former	7 (14.6)
Never	41 (85.4)
Eastern Cooperative Oncology Group, *n* (%)	
0	15 (31.3)
1	33 (68.8)
Previous treatment therapies, *n* (%)	
0	25 (52.1)
1	14 (29.2)
≥2	9 (18.8)
Previous chemotherapy, *n* (%)	
Yes	21 (43.8)
No	27 (56.3)
Previous immunotherapy, *n* (%)	
Yes	16 (33.3)
No	32 (66.7)
HER2 mutations, *n* (%)	
Insertion mutation	41 (85.4)
Missense mutation	6 (12.5)
HER2 amplification	1 (2.1)
TP53 mutation status, *n* (%)	
Positive	27 (56.3)
Negative	18 (37.5)
Unknown	3 (6.3)

### 2.2 Safety

All 48 patients experienced treatment‐related toxicities. No dose‐limiting toxicity (DLT) occurred during escalation period. Hence, inetetamab with 320 mg pyrotinib was determined as expansion scheme. The most common TRAEs included diarrhea (66.7% [2/3] in inetetamab with 240 mg pyrotinib group, 95.6% [43/45] in inetetamab with 320 mg pyrotinib group), rash (66.7% [2/3] in inetetamab with 240 mg pyrotinib group, 22.2% [10/45] in inetetamab with 320 mg pyrotinib group), and vomiting (0 inetetamab with 240 mg pyrotinib group, 24.4% [11/45] in inetetamab with 320 mg pyrotinib group) (Table 2). TRAEs were generally grade (G) 1–2. G3 TRAEs occurred in seven patients (one in inetetamab with 240 mg pyrotinib group, six in inetetamab with 320 mg pyrotinib group), with diarrhea and increased alanine transaminase (ALT) being the most common events (Table S1). No G4 or higher TRAEs were reported. As for pulmonary toxicity, one G2 and one G3 pneumonia were documented. Eight patients experienced dose reduction or discontinuation due to TRAEs. Four patients discontinued treatment due to G3 TRAEs (one G3 pneumonia in inetetamab with 240 mg pyrotinib group, two G3 diarrhea, and one G3 increased ALT/aspartate aminotransferase [AST] in inetetamab with 320 mg pyrotinib group). The detailed information of dose reduction and treatment discontinuation events is documented in Table S2.

**TABLE 2:** Most common treatment‐related adverse events in the study population.

Event	Grade 1	Grade 2	Grade 3	Overall
Treatment‐related adverse events	40 (83.3)	19 (39.6)	7 (14.6)	46 (95.8)
Occurring in ≥10% of patients				
Diarrhea	29 (60.4)	14 (29.2)	2 (4.2)	45 (93.8)
Rash	11 (22.9)	1 (2.1)	0	12 (25.0)
Vomiting	9 (18.8)	2 (4.2)	0	11 (22.9)
Blood creatinine increased	9 (18.8)	0	1 (2.1)	10 (20.8)
Blood uric acid increased	10 (20.8)	0	0	10 (20.8)
Paronychitis	7 (14.6)	1 (2.1)	1 (2.1)	9 (18.8)
Anemia	9 (18.8)	0	0	9 (18.8)
Nausea	8 (16.7)	0	0	8 (16.7)
Decreased appetite	7 (14.6)	1 (2.1)	0	8 (16.7)
PPE syndrome	5 (10.4)	0	0	5 (10.4)
Dizziness	4 (8.3)	1 (2.1)	0	5 (10.4)
Cough	4 (8.3)	1 (2.1)	0	5 (10.4)
Chest pain	4 (8.3)	1 (2.1)	0	5 (10.4)
Back pain	5 (10.4)	0	0	5 (10.4)

Abbreviation: PPE, Palmar‐Plantar Erythrodysesthesia.

### 2.3 Efficacy

**FIGURE 2:**
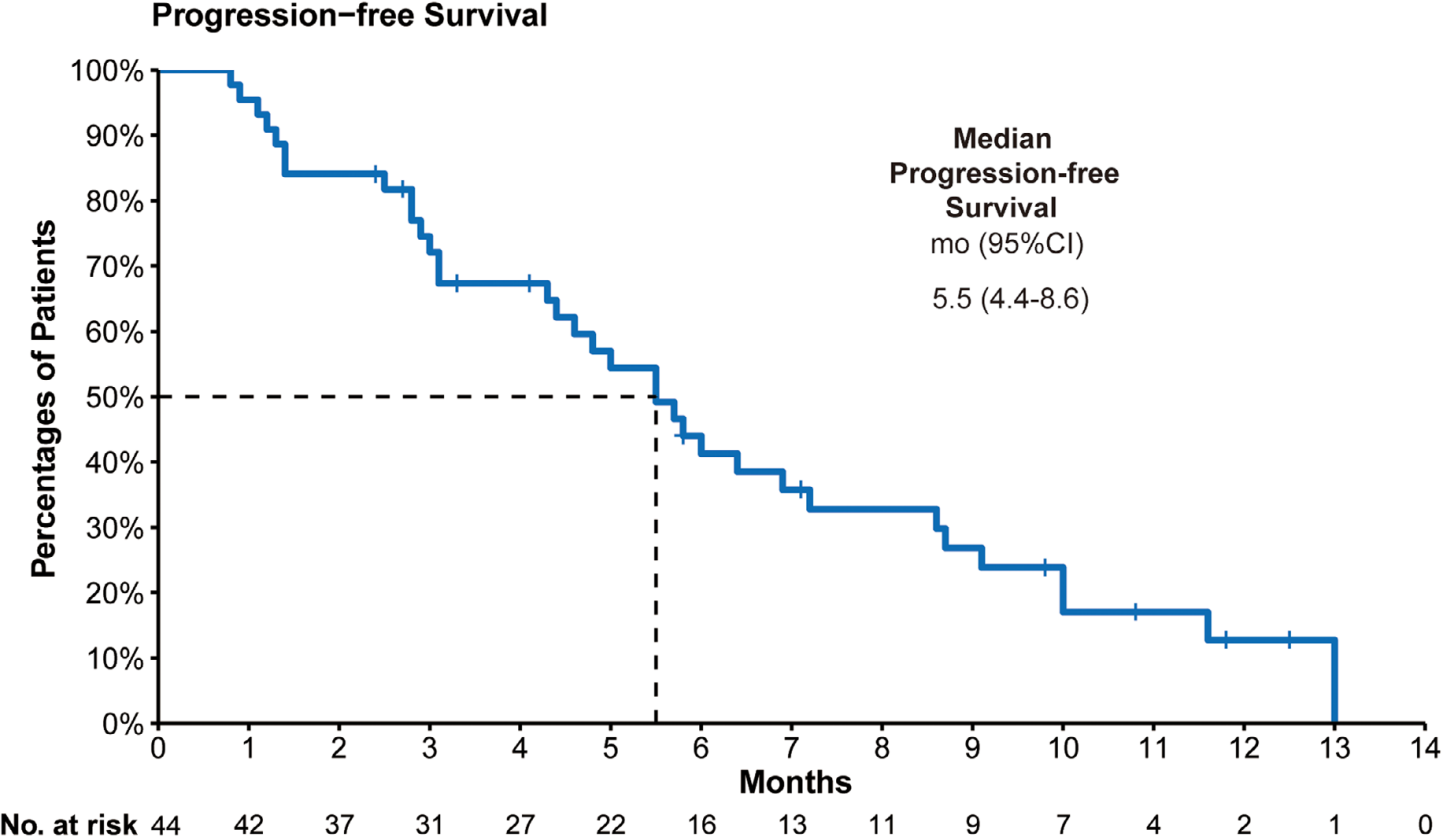
Kaplan–Meier survival curves of PFS in 44 patients receiving inetetamab in combination with pyrotinib. PFS, progression‐free survival; CI, confidence interval; mo, month/s.

**FIGURE 3:**
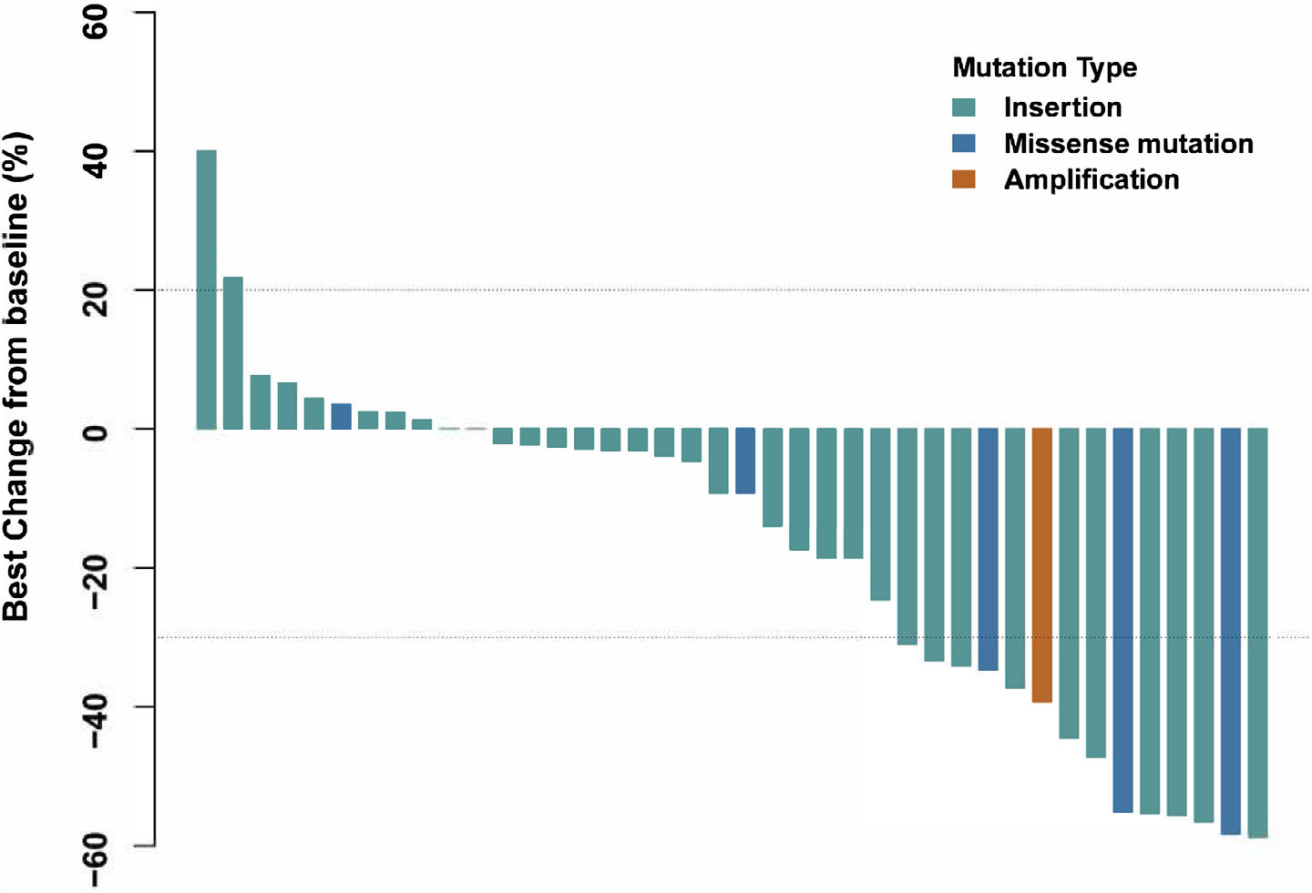
Waterfall plot of best response. A total of 40 patients with evaluable tumor change were included.

A total of 44 patients were included for efficacy assessments (three in inetetamab with 240 mg pyrotinib group, 41 in inetetamab with 320 mg pyrotinib group). As of March 20, 2023, 34 PFS events occurred, six patients discontinued without disease progression, and four patients continued to receive combination therapies. The mPFS was 5.5 months [95% CI: 4.4–8.6 months] (Figure 2). The confirmed ORR and disease control rate (DCR) reached 25% (11/44) and 84.1% (37/44), respectively. The mPFS of 41 patients receiving inetetamab with 320 mg pyrotinib was 5.5 months [95% CI: 4.4–8.6 months] (Figure S1). Among the inetetamab with 240 mg pyrotinib group, two stable disease (SD) and one progressive disease (PD) were seen (confirmed ORR 0, DCR 66.7%). Eleven patients had a confirmed partial response (PR), and 35 achieved disease control in inetetamab with 320 mg pyrotinib group (confirmed ORR 26.8%, DCR 85.4%) (Table 3). Tumor size changes from baseline over time are depicted in Figure 3.

**TABLE 3:** Clinical response to inetetamab with pyrotinib in NSCLC patients with HER2 mutations.

Variable	
Best response, *n* (%)	
Inetetamab with 240 mg pyrotinib (*n* = 3)	
Confirmed partial response	0 (0)
Stable disease	2 (66.7)
Progressive disease	1 (33.3)
Inetetamab with 320 mg pyrotinib (*n* = 41)	
Confirmed partial response	11 (26.8)
Stable disease	24 (58.5)
Progressive disease	6 (14.6)
Confirmed objective response rate, %	
Inetetamab with 240 mg pyrotinib	0
Inetetamab with 320 mg pyrotinib	26.8
Disease control rate, %	
Inetetamab with 240 mg pyrotinib	66.7
Inetetamab with 320 mg pyrotinib	85.4
Progression‐free survival	
Events, *n* (%)	34 (77.3)
Median, months [95% CI]	5.5 [4.4–8.6]

**FIGURE 4:**
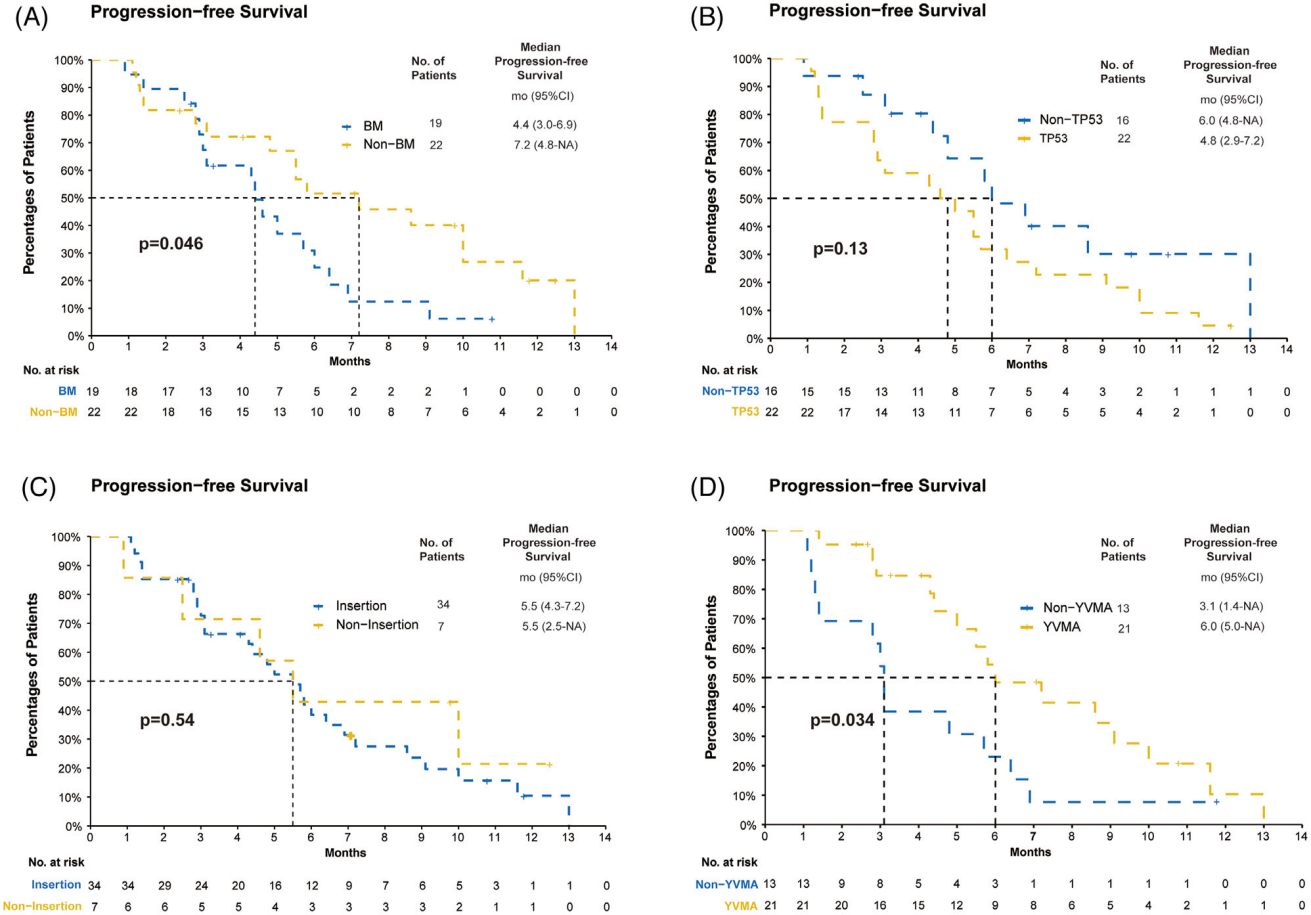
Subgroup analysis of Kaplan–Meier survival curves of PFS in patients receiving inetetamab in combination with 320 mg pyrotinib. (A) BM subgroup analysis. (B) TP53 status subgroup analysis. (C) Mutation type subgroup. (D) Insertion type subgroup. BM, brain metastasis; PFS, progression‐free survival; CI, confidence interval; mo, month/s.

Subgroup analyses were conducted in patients receiving inetetamab with 320 mg pyrotinib. The patients receiving inetetamab with pyrotinib as first‐line treatment showed a comparable mPFS (mPFS, 5.5 vs. 5.5 months, *p* = 0.81) and better ORR (ORR 40.9% [9/22] vs. 10.5% [2/19], *p* = 0.038) than patients with prior therapies. Similar DCR was observed (DCR 86.4% [19/22] vs. 84.2% [16/19], *p* = 1). Prior chemotherapy and immunotherapy‐based treatment did not affect the ORR and PFS outcomes. Patients with brain metastases at baseline tended to have inferior PFS (mPFS 4.4 vs. 7.2 months, *p* = 0.046; Figure 4A) and worse ORR (ORR 15.8% [3/19] vs. 36.4% [8/22], *p* = 0.17) than those without brain metastases. TP53 status was detected in 38 patients receiving 320 mg pyrotinib, and 57.9% (22/38) had TP53 aberrations. Patients without TP53 mutations tended to have a longer PFS than those with TP53 mutations (mPFS 6.0 vs. 4.8 months, *p* = 0.13; Figure 4B). No significant difference was observed in ORR regarding TP53 status. Baseline clinical features (sex, age, smoking status, ECOG) did not significantly affect ORR or PFS. The detailed results of subgroup analyses are concluded in Table S3 and Figure S2.

Responses were observed in patients with various HER2 aberrations including HER2 exon 20 insertion mutations (ORR 20.6% [7/34]), missense mutations (ORR 50% [3/6]), and HER2 amplification (one PR) (Table S4). The confirmed ORR achieved by patients with HER2 Y772_A775 dup and G776delinsVC were 28.6% (6/21) and 16.7% (1/6), respectively. No PR was seen in six patients with HER2 G778_P780dup and one patient with G778_S779insYPG (Table S4). Among six patients with HER2 missense mutations, three patients with HER2 S310F, V659D, or S310Y/D769H achieved PR, two patients with HER2 I767M or V777L achieved SD and one patient with HER2 V659E achieved PD. The mPFS of patients with HER2 missense mutations was 5.1 months [95% CI: 2.5 to NA months]. The patient with HER2 amplification achieved PR with a long PFS of over 12.5 months (treatment ongoing). There existed no significant difference in ORR and PFS between patients with HER2 exon 20 insertion and non‐insertion mutations (ORR 20.6% [7/34] vs. 57.1% [4/7], *p* = 0.069; mPFS 5.5 vs. 5.5 months, *p* = 0.54; Figure 4C). Upon dissection by HER2 insertion types, it is noteworthy that patients with HER2 Y772_A775 dup achieved a higher ORR (ORR 28.6% [6/21] vs. 7.7% [1/13], *p* = 0.017) and longer PFS (mPFS 6.0 vs. 3.1 months, *p* = 0.034; Figure 4D) than patients with non‐YVMA insertion mutations.

## 3 DISCUSSION

To date, the exploration of new targeted therapies remains an urgent need for advanced HER2‐mutant NSCLC due to a lack of effective anti‐HER2 inhibitors, with only traditional chemotherapies and T‐DXd being approved. Despite the development of dual HER2‐targeted therapies in breast cancer, related studies on lung cancer are lacking. In the MyPathway trial, anti‐HER2 monoclonal antibodies trastuzumab in combination with pertuzumab reported a 21% [95% CI: 5%–51%] ORR for patients with NSCLC (*n* = 12).^25^ However, a modest antitumor activity of this combination therapy was reported in a larger prospective cohort (*n* = 24), with an ORR of 8.3% and an mPFS of 4 months.^26^ The development of dual targeted therapies in HER2‐mutant lung cancer patients still demands more effort.

Among novel HER2 TKIs, pyrotinib showed moderate clinical benefit with a manageable toxicity profile.^13^, ^14^ Pyrotinib covalently binds to the ATP binding site of the intracellular kinase domain of HER1, HER2, and HER4.^10^ Inetetamab is an anti‐HER2 monoclonal antibody binding to domain IV of HER2 receptor.^19^ The addition of inetetamab may exert more potent inhibition of HER2 signaling pathway via dual HER2 blockade. Besides, inetetamab, an analog of trastuzumab, was more capable of stimulating antibody‐dependent cell‐mediated cytotoxicity (ADCC) effect with a modified Fc segment.^23^, ^27^ Hence, a robust antitumor activity may be induced by combining a monoclonal antibody, which may mediate more powerful immune response and receptor downregulation.^28^, ^29^, ^30^ Multiple inhibitory effects induced by the two drugs provide the rationale for evaluating the antitumor activity of the combination therapy.

In this clinical trial, we aimed to evaluate the safety and efficacy of the dual HER2‐targeted therapy consisting of inetetamab and pyrotinib (240 mg, 320 mg) in advanced NSCLC patients with HER2 mutations. No DLT occurred in dose‐escalation period. The most common TRAEs were diarrhea, rash, and vomiting. Seven patients with G3 TRAEs were documented, and no G4 or higher TRAEs occurred. The confirmed ORR and mPFS reached 25% and 5.5 months [95% CI: 4.4–8.6 months], respectively. The patients receiving inetetamab with 320 mg pyrotinib reached a higher ORR of 26.8%. Our study preliminarily showed manageable toxicity and clinical benefit of inetetamab plus pyrotinib in HER2‐mutant patients with advanced NSCLC, providing a promising combination therapy deserving further study.

In a previous clinical trial, 28.3% of NSCLC patients receiving pyrotinib at 400 mg had G3–G4 TRAEs that commonly manifested as diarrhea (20%), which resulted in dose interruptions and treatment discontinuations in 21.7% and 1.7% of patients, respectively.^13^ As for patients receiving recently approved T‐DXd, 46% of patients had G3 or higher TRAEs, with drug‐related interstitial lung disease occurring in 26% of patients.^16^ In our study, a slightly lower incidence of G3 TRAEs (14.6%) was documented, and 16.7% of patients experienced dose reduction or treatment discontinuation. Only two pneumonia cases were documented, showing a relatively low pulmonary toxicity compared with T‐DXd. A manageable safety profile of this combination regimen was shown.

As the data showed, inetetamab combined with pyrotinib at a relatively low dose (240 and 320 mg) showed a comparable ORR (25%) and mPFS (5.5 months, 95% CI: 4.4–8.6 months) with patients receiving 400 mg pyrotinib in previous studies (Song et al.’s study: ORR 19.2%, mPFS 5.6 months; Zhou et al.’s study: ORR 30%, mPFS 6.9 months).^13^, ^14^ Most patients (84.1%) in this study achieved disease control. The comparable efficacy achieved by this dual blockade therapy might result from the relatively lower dose of pyrotinib in our study (240 and 320 mg) than that in other clinical trials (400 mg). In addition, a higher percentage of patients with brain metastases was enrolled in this trial (50% in our study vs. 25.6% in Song et al.’s study and 20% in Zhou et al.’s study).^13^, ^14^ The insufficient capability to penetrate the blood–brain barrier of TKI at lower dose might restrict the antitumor activity of the combination therapy. Patients with brain metastases at baseline in this study did have worse PFS and ORR than those without brain metastases. In short, inetetamab with pyrotinib, albeit at a clinically lower dose, still showed a positive antitumor activity.

Antitumor activity of the combination strategy was observed across diverse HER2 mutation subtypes. There existed no significant difference in ORR or PFS between patients with insertion mutations and non‐insertion mutations, indicating the broad inhibiting activity of the combination therapy. Prior studies have shown that among HER2 exon 20 insertion mutations, HER2 Y772_A775 dup was less responsive to TKIs.^8^, ^12^, ^13^, ^31^ It is important to explore better approaches to improving clinical activity of this subtype because HER2 Y772_A775 dup is the most common HER2 exon 20 insertion mutation. In our study, we found that patients with HER2 Y772_A775 dup achieved a higher ORR (28.6% vs. 7.7%, *p* = 0.017) and longer mPFS (6.0 vs. 3.1 months, *p* = 0.034) than patients with non‐YVMA insertion mutation, which implied that adding a monoclonal antibody might enhance the sensitivity to pyrotinib in this less‐responsive population. Similar results were observed in patients with EGFR exon 20 insertion mutations. Previous studies on EGFR exon 20 insertion mutations, which were resistant to most EGFR TKIs, also demonstrated that adding an EGFR antibody could improve the sensitivity to EGFR TKIs.^30^, ^32^, ^33^ More sustained EGFR blockade via signaling inhibition and receptor downregulation by the combination therapy might explain the improved antitumor activity.^30^ Although the underlying mechanisms of the superior efficacy in patients with HER2 Y772_A775 dup were unclear, our results strongly indicated a promising approach for this major subgroup of patients.

The current study has some limitations. The sample was small because of the low prevalence of HER2 aberrations in lung cancer. Only one patient with HER2 amplification was included. The design of single arm disabled the comparison of clinical benefits between the combination regimen and other therapies. Larger randomized clinical trials are in demand to warrant the findings in our study. We did not investigate the underlying mechanisms of the combination therapy, which deserved further studies. Lastly, TMB data were not available for subgroup analysis, because of limited TMB tests and inconsistent NGS methods.

In summary, our study preliminarily showed the manageable safety profile and promising antitumor activity of inetetamab combined with pyrotinib in advanced NSCLC harboring HER2 aberrations.

## 4 METHODS

### 4.1 Patients

Patients were recruited at Sun Yat‐sen University Cancer Center from August 2021 to August 2022. Eligible patients were advanced NSCLC (stage IIIB or IV) harboring HER2 aberrations. Next‐generation sequencing (NGS) was applied to identify HER2 mutations. Other inclusion criteria were age 18 years or older, ECOG performance‐status score of 0–1, measurable disease by Response Evaluation Criteria in Solid Tumors (RECIST) version 1.1.

Patients who had previously been treated with anti‐HER2 monoclonal antibody therapies were ineligible for participation. Patients with previous surgery, radiotherapy, chemotherapy, or any other targeted therapy for NSCLC within 4 weeks prior to the initiation of the combination therapy were also excluded.

All patients provided written informed consent before starting any trial‐related treatment. The study protocol was approved by Sun Yat‐sen University Cancer Center IRB (B2021‐188) according to the principles of the Declaration of Helsinki and Good Clinical Practice guidelines.

### 4.2 Study design and treatment

This opening, dose‐escalating phase Ib clinical trial was conducted at Sun Yat‐sen University Cancer Center in China. HER2‐positive patients with advanced NSCLC were enrolled to receive anti‐HER2 antibody inetetamab combined with pan‐HER inhibitor pyrotinib. The study comprised two parts, dose‐escalation group and dose‐expansion group. During the dose‐escalation period, the parallel “3+3” dose‐escalation design was performed to determine the DLT (defined as any G3–G4 adverse events related to treatment during the first cycle). The combination scheme with well‐tolerable safety profiles would be selected for the dose‐expansion part to further explore the safety and efficacy. In the early clinical trial of pyrotinib, the maximum tolerated dose was identified as 400 mg.^34^ In view of the possible additive toxicity caused by two anti‐HER2 drugs, pyrotinib 320 mg was determined as the highest escalation dose in this study. Inetetamab was administered intravenously once every 3 weeks (loading dose of 8 mg/kg and subsequent doses of 6 mg/kg) with pyrotinib orally once per day (dose‐escalation part: 240 mg, 320 mg; dose‐expansion part: 320 mg). NGS tests were used to analyze the biopsy tissue samples or blood samples obtained from enrolled patients.

### 4.3 Safety and efficacy assessment

Primary endpoints were DLT dosage and TRAEs. The DLT was evaluated within 21 days after the initiation of first cycle. Adverse events were assessed with the application of the Common Terminology Criteria for Adverse Events (CTCAE), version 5.0. The secondary endpoints included confirmed ORR, DCR assessed by independent central review in accordance with RECIST, version 1.1, and PFS (time between the initiation of the combination treatment and disease progression or any‐cause death occurrence). Tumor imaging assessments were carried out every 6 weeks.

### 4.4 Statistical analysis

All statistical analyses were conducted with GraphPad prism (version 9) and SPSS statistical software (version 20.0). PFS and OS were analyzed using Kaplan–Meier curves, with *p*‐value determined by a log‐rank test. The ORR and DCR differences between different subgroups were assessed using the Fisher's exact test. Hazard ratio (HR) and 95% CIs were calculated with Cox regression. A two‐tailed *p*‐value less than 0.05 was considered statistically significant.

## AUTHOR CONTRIBUTIONS

Study concept and design: Wenfeng Fang, Li Zhang, Yihua Huang, Yuanyuan Zhao, Yan Huang, Yunpeng Yang, and Yaxiong Zhang. Data acquisition and analysis: all authors. Data interpretation: Yihua Huang, Yuanyuan Zhao, Yan Huang, Yunpeng Yang, and Yaxiong Zhang. Manuscript drafting: Yihua Huang, Yuanyuan Zhao, Yan Huang, Yunpeng Yang, and Yaxiong Zhang. Critical revision of the manuscript: Wenfeng Fang, Li Zhang, and Yihua Huang. Statistical analysis: Wenfeng Fang, Li Zhang, and Yihua Huang. All authors have read and approved the final manuscript.

## CONFLICT OF INTEREST STATEMENT

The authors declare they have no conflicts of interest.

## ETHICS STATEMENT

Clinical trial registration: ClinicalTrial.gov: NCT05016544. The study protocol was approved by Sun Yat‐sen University Cancer Center IRB (B2021‐188) in accordance with the Declaration of Helsinki and Good Clinical Practice guidelines.

## Supporting information

Supporting Information

## Data Availability

The datasets used and/or analyzed during the current study are available from the corresponding author upon reasonable request.
