# Supplementary material for: Phase 1b trial of anti‐HER2 antibody inetetamab and pan‐HER inhibitor pyrotinib in HER2‐positive advanced lung cancer
Source: MedComm (2020). 2024 Apr 29;5(5):e536. doi: 10.1002/mco2.536 (PMC11057420; doi:10.1002/mco2.536)
Supplement: Supplementary file 1 — Supporting Information [file MCO2-5-e536-s001.docx]

**Phase 1b trial of anti-HER2 antibody inetetamab and pan-HER inhibitor pyrotinib in HER2-positive advanced lung cancer**

**Running title:** Inetetamab with Pyrotinib in HER2-positive NSCLC.

**Authors and Affiliations**

Yihua Huang^1#^, Yuanyuan Zhao^1#^, Yan Huang^1#^, Yunpeng Yang^1#^, Yaxiong Zhang^1#^, Shaodong Hong^1^, Hongyun Zhao^2^, Shen Zhao^1^, Ting Zhou^1^, Gang Chen^1^, Huaqiang Zhou^1^, Yuxiang Ma^2^, Ningning Zhou^1^, Li Zhang^1^*, Wenfeng Fang^1^*

^1^Department of Medical Oncology, State Key Laboratory of Oncology in South China, Guangdong Provincial Clinical Research Center for Cancer, Collaborative Innovation Center for Cancer Medicine, Sun Yat-Sen University Cancer Center, Guangzhou 510060, People’s Republic of China

^2^Department of Clinical Research, State Key Laboratory of Oncology in South China, Guangdong Provincial Clinical Research Center for Cancer, Collaborative Innovation Center for Cancer Medicine, Sun Yat-sen University Cancer Center, Guangzhou, China.

#These authors contributed equally to this work.

***Correspondence:**

Li Zhang, Address for correspondence: Department of Medical Oncology, State Key Laboratory of Oncology in South China, Guangdong Provincial Clinical Research Center for Cancer, Collaborative Innovation Center for Cancer Medicine, Sun Yat-Sen University Cancer Center, Guangzhou 510060, People’s Republic of China. E-mail: [zhangli6@mail.sysu.edu.cn](mailto:zhangli6@mail.sysu.edu.cn)

Wenfeng Fang, Address for correspondence: Department of Medical Oncology, State Key Laboratory of Oncology in South China, Guangdong Provincial Clinical Research Center for Cancer, Collaborative Innovation Center for Cancer Medicine, Sun Yat-Sen University Cancer Center, Guangzhou 510060, People’s Republic of China. E-mail: [fangwf@sysucc.org.cn](mailto:fangwf@sysucc.org.cn).

**Supplementary figure 1.** Kaplan-Meier survival curves of PFS in 41 patients receiving inetetamab in combination with 320mg pyrotinib. PFS, progression-free survival; 95%CI, 95% confidence interval; mo, month.


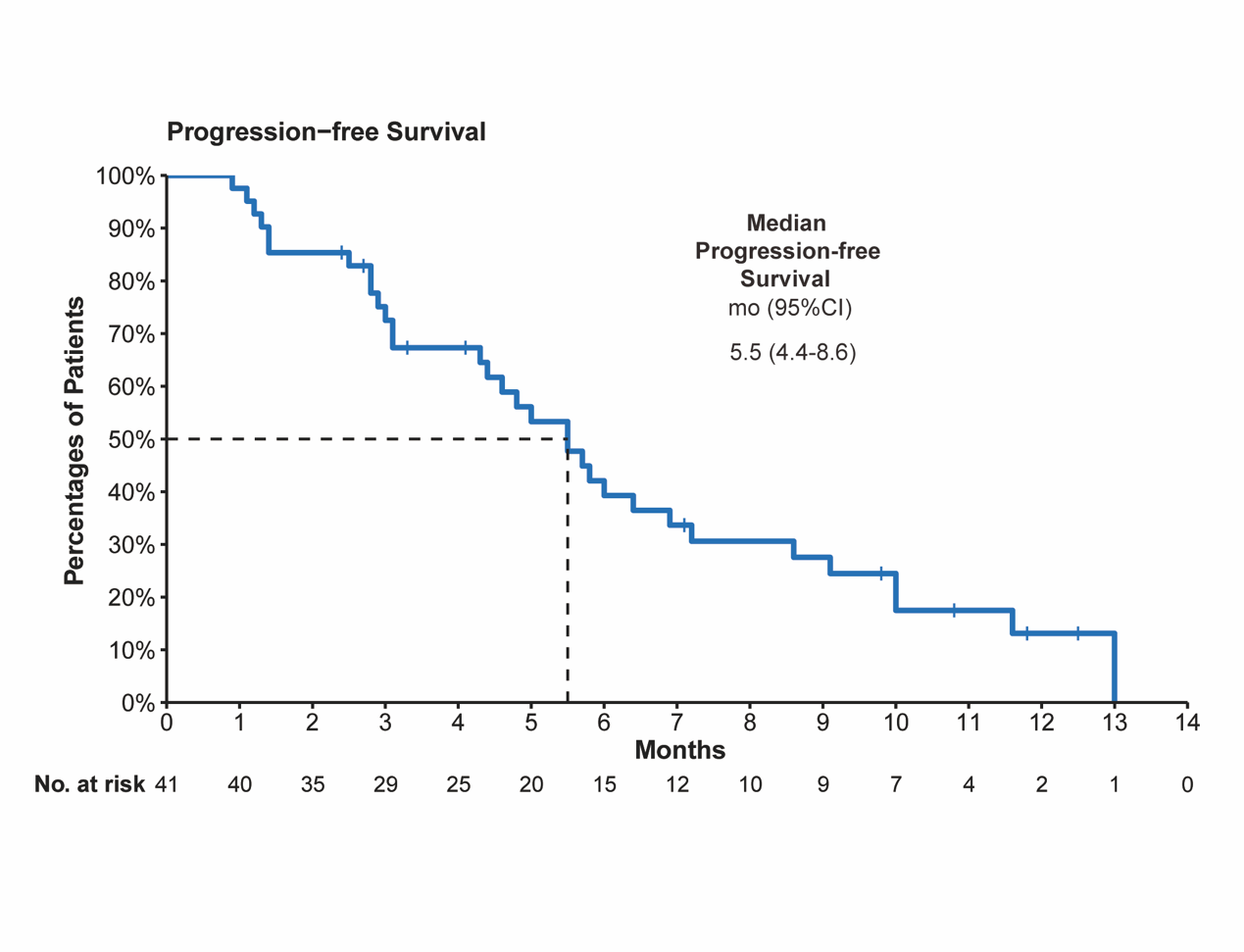


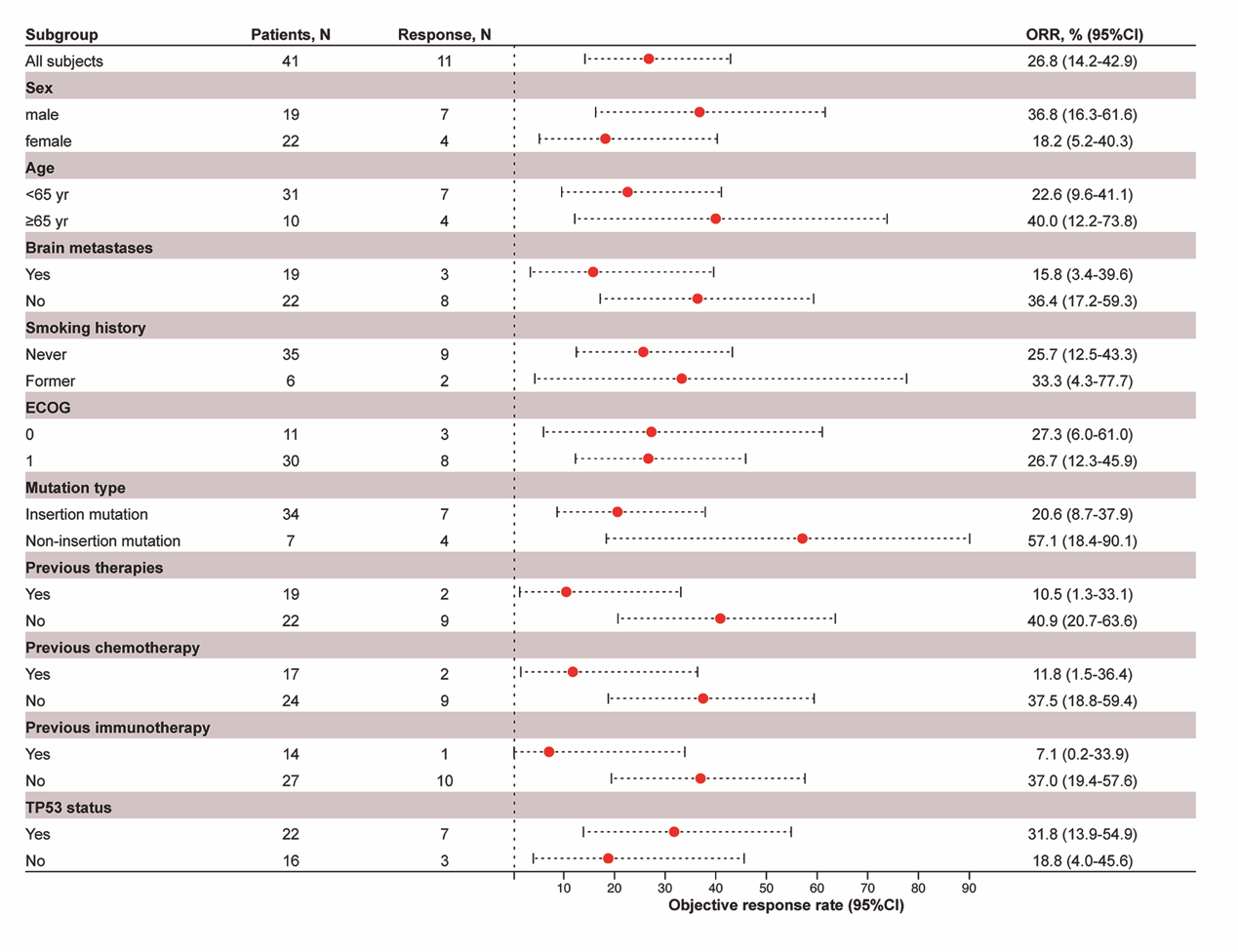
**Supplementary figure 2.** ORR subgroup analysis of patients receiving inetetamab in combination with 320mg pyrotinib. ORR, objective response rate.

**Supplementary table 1. Grade 3 treatment-related adverse events.**

| **Patient** | **Grade 3 TRAEs** |
| --- | --- |
| 1 | Pneumonia |
| 6 | Diarrhea |
| 7 | Paronychitis |
| 15 | Increased ALT |
| 25 | Increased ALT/AST |
| 33 | Infusion related reaction |
| 46 | Diarrhea/increased blood creatinine |

TRAEs, treatment-related adverse events. ALT, alanine transaminase. AST, aspartate aminotransferase

**Supplementary table 2. Detailed information of treatment discontinuation and dose reduction.**

| **Patient** | **Related TRAEs** | **Dose alteration** | |
| --- | --- | --- | --- |
| 1 | G3 pneumonia | | Treatment discontinuation (withdrawal due to SAE) |
| 6 | G3 diarrhea | | Pyrotinib 320mg to 240mg (withdrawal due to SAE) |
| 7 | G3 paronychitis | | Pyrotinib discontinuation (withdrawal due to lumbar disease) |
| 25 | G3 increased ALT/AST | | Treatment discontinuation (withdrawal due to SAE) |
| 29 | G2 decreased appetite | | Pyrotinib 320mg to 160mg, 80mg |
| 33 | G3 Infusion related reaction | | Treatment discontinuation (withdrawal due to cerebral infarction) |
| 45 | G2 diarrhea | | Pyrotinib 320mg to 240mg |
| 46 | G3 diarrhea/increased blood creatinine | | Pyrotinib 320mg to 240mg (withdrawal due to SAE without response assessment) |

G, grade; SAE, serious adverse events.

**Supplementary table 3. PFS subgroup analysis of patients receiving inetetamab with 320mg pyrotinib.**

| **Parameter** | **No. of Patients** | **mPFS (mo)** | **PFS HR (95% CI)** | **p** |
| --- | --- | --- | --- | --- |
| **Overall** | 41 |  |  |  |
| **Sex** |  |  |  |  |
| Male | 19 | 5.8 | 0.76(0.37-1.56) | 0.44 |
| Female | 22 | 5.0 |  |  |
| **Age at screening** |  |  |  |  |
| <65 yr | 31 | 5.7 | 0.64 (0.30-1.36) | 0.25 |
| ≥65 yr | 10 | 5.1 |  |  |
| **Brain metastases** |  |  |  |  |
| Yes | 19 | 4.4 | 2.07 (0.99-4.33) | 0.046 |
| No | 22 | 7.2 |  |  |
| **Smoking history** |  |  |  |  |
| Never | 35 | 5.5 | 0.78 (0.30-2.05) | 0.63 |
| Former | 6 | 4.8 |  |  |
| **ECOG** |  |  |  |  |
| 0 | 11 | 6.4 | 0.72 (0.31-1.67) | 0.43 |
| 1 | 30 | 5.5 |  |  |
| **Mutation type** |  |  |  |  |
| Insertion mutation | 34 | 5.5 | 1.33 (0.51-3.49) | 0.54 |
| Non-Insertion mutation | 7 | 5.5 |  |  |
| **Previous therapies** |  |  |  |  |
| Yes | 19 | 5.5 | 1.09 (0.53 -2.21) | 0.81 |
| No | 22 | 5.5 |  |  |
| **Previous chemotherapy** |  |  |  |  |
| Yes | 17 | 5.5 | 1.05 (0.50-2.17) | 0.88 |
| No | 24 | 5.5 |  |  |
| **Previous immunotherapy** |  |  |  |  |
| Yes | 14 | 5.0 | 1.04 (0.49 -2.22) | 0.91 |
| No | 27 | 5.8 |  |  |
| **TP53 status** |  |  |  |  |
| Negative | 16 | 6.0 | 0.54 (0.25-1.19) | 0.13 |
| Positive | 22 | 4.8 |  |  |

PFS, progression-free survival; HR, hazard ratio; mo, months.

**Supplementary table 4. Clinical response to inetetamab in combination with 320mg pyrotinib in NSCLC patients with different HER2 mutations**

| **Mutation subtype** | **Insertion (n=34)** | | | | **Missense mutation (n=6)** | **HER2 amplification**  **(n=1)** |
| --- | --- | --- | --- | --- | --- | --- |
|  | **Y772_A775 dup (n=21)** | **G776delinsVC (n=6)** | **G778_P780dup (n=6)** | **G778_S779insYPG (n=1)** |  |  |
| **Best Response, n (%)** |  |  |  |  |  |  |
| Confirmed partial response | 6 | 1 | 0 | 0 | 3 | 1 |
| Stable disease | 14 | 2 | 5 | 1 | 2 | 0 |
| Progressive disease | 1 | 3 | 1 | 0 | 1 | 0 |
| **Confirmed ORR, %** | 28.6 | 16.7 | 0 | 0 | 50.0 | 100 |
| **DCR, %** | 95.2 | 50.0 | 83.3 | 100 | 83.3 | 100 |

ORR, objective response rate, DCR, disease control rate.
